# A groom with a view

**DOI:** 10.7554/eLife.88595

**Published:** 2023-05-16

**Authors:** Jeffrey E Markowitz

**Affiliations:** 1 Wallace H. Coulter Department of Biomedical Engineering, Georgia Institute of Technology and Emory University Atlanta United States

**Keywords:** innate naturalistic behavior, self-grooming, striatum, innate behavior, Mouse

## Abstract

Mapping mouse grooming episodes to neural activity shows that striatal cells deep in the brain collectively represent key aspects of self-grooming.

**Related research article** Minkowicz S, Mathews MA, Mou FH, Yoon H, Freda SN, Cui ES, Kennedy A, Kozorovitskiy Y. 2023. Striatal ensemble activity in an innate naturalistic behavior. *eLife* **12**:RP87042. doi: 10.7554/eLife.87042.

Are you sitting still right now, or are you moving? Occasionally, our brains compel us to scratch an itch, comb our fingers through our hair, or stroke our chin without even thinking about it. These actions may seem inconsequential to us, but they are examples of a unique type of behavior called self-grooming. Found in species spanning most of the animal kingdom – from monkeys to prairie voles and even fruit flies – self-grooming can serve as a powerful lens for revealing what the brains of monkeys and fruit flies might have in common.

Self-grooming is commonly defined as a behavior used to care for the outside of the body (Spruijt et al., 1992). While the particulars of a mouse groom may look dramatically different to those of a fruit fly, they both involve a highly stereotyped sequence of behaviors, such as rubbing the digits together followed by touching the face in elliptical strokes (Fentress and Stilwell, 1973; Szebenyi, 1969). This suggests that the brains of multiple species may share a common neural mechanism that controls how behaviors are organized in time, or ‘sequenced’. A single brain circuit – the basal ganglia, a conserved set of neurons deep within the brain – appears to sequence these actions. Pioneering work showed that introducing lesions or other perturbations to the striatum – which is part of the basal ganglia – disrupts the stereotypical sequence in which self-grooming behaviors occur, confirming that the striatum has a key role in the process (Berridge and Whishaw, 1992; Cromwell and Berridge, 1996; Van den Bercken and Cools, 1982). Recordings from striatal neurons showed that they responded during different phases of grooming episodes (Aldridge and Berridge, 1998). However, it remained unclear how the neurons within the striatum work together to produce the sequence of self-grooming.

Now, in eLife, Ann Kennedy and Yevgenia Kozorovitskiy of Northwestern University and colleagues – including Samuel Minkowicz as first author – report a new method for identifying grooming episodes in video footage, and then show that discrete groups of striatal neurons show activity at the beginning and end of such episodes in mice, as well as throughout episodes (Minkowicz et al., 2023).

Historically, grooming episodes were identified by hand-labeling high-speed videos (Fentress and Stilwell, 1973), which was time-consuming and also limited the scale of studies. Complementing recent developments in automated identification of grooming episodes from raw video footage (Geuther et al., 2021), Minkowicz et al. developed a semi-automated technique to algorithmically identify mouse grooming episodes based on the movement of key body parts in 3D. Detecting certain movements – such as a mouse moving its paws close to its nose – helped to isolate grooming episodes from over 100 hours of video footage. Furthermore, the code used by Minkowicz et al. is open-source, which will allow the scientific community to benefit from this new method.

In addition to capturing video footage, Minkowicz et al. used probes embedded in the striatum of mice to record neuronal activity and investigate how neurons in the striatum collectively represent grooming. The recordings showed that when a mouse is relatively still, striatal cells fire sparsely and randomly. However, during a grooming episode, the cells appear to coalesce into clusters of neurons that fire simultaneously, in line with recent recordings of striatal neurons during other types of movement (Klaus et al., 2017; Barbera et al., 2016). These ‘ensembles’ do not represent grooms with perfect fidelity – if a cell fires during one bout of grooming, it is not guaranteed to fire during the next. However, through studying a large number of grooms, it was clear that ensembles of striatal neurons become active at key stages of grooming episodes such as the beginning and the end, or for the duration of the episode (Figure 1).

**Figure 1.**
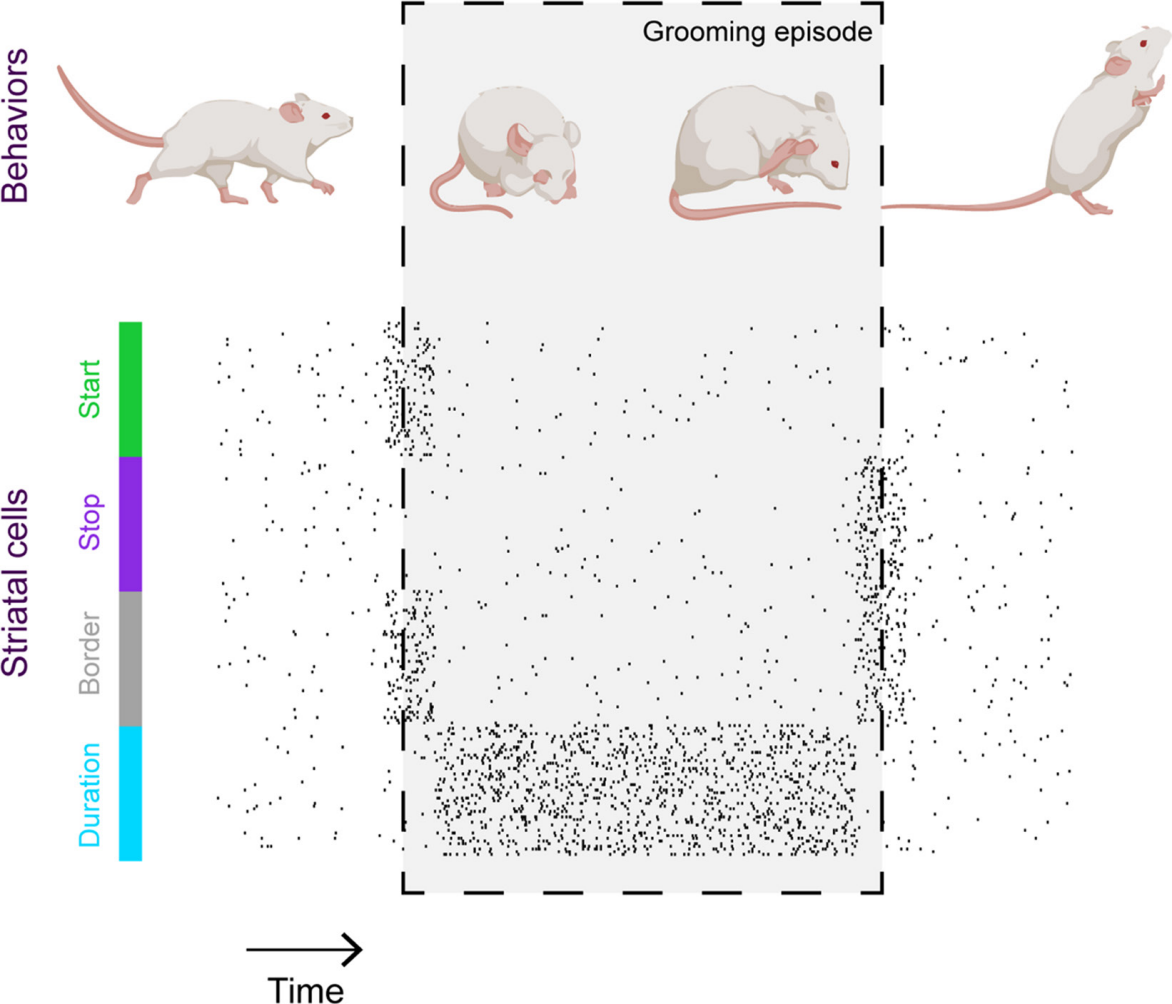
Striatal activity collectively represents key features of mouse grooming. Mouse grooming episodes (depicted within box) involve certain behaviors such as rubbing the eyes and ears with paws. By simultaneously recording mouse behavior and neural activity in the striatum – a key brain structure known to be involved in sequencing behavior – Minkowicz et al. showed that the activity of striatal cells is orchestrated to represent the important timepoints in a grooming sequence: when grooming starts (green); when grooming stops (purple); at both the start and end of the grooming episode (‘border’, grey) and for the duration of the episode (blue).

Image credit: Mouse cartoons created with BioRender.

The findings suggest that grooming, and perhaps other spontaneous behaviors, are represented by small ensembles of striatal neurons as discrete, unitary objects that are strung together to form sequences. This is potentially at odds with other work that has found that the striatum represents continuous aspects of movement like velocity and vigor (Panigrahi et al., 2015; Dhawale et al., 2021), although these possibilities are not mutually exclusive. Moving forward, new experimental and computational approaches will be required to resolve the question at the heart of basal ganglia function: does it assemble behavioral sequences from a set of discrete puzzle pieces, or do sequences emerge through the direct control of continuous aspects of motor control?
